# Fabrication and Fireproofing Performance of the Coal Fly Ash-Metakaolin-Based Geopolymer Foams

**DOI:** 10.3390/ma13071750

**Published:** 2020-04-09

**Authors:** Xi Peng, Qin Shuai, Han Li, Qin Ding, Yan Gu, Chunjie Cheng, Zhonghui Xu

**Affiliations:** 1 State Key Laboratory of Fire Science, University of Science and Technology of China, Hefei 230026, China; pengxi@mail.ustc.edu.cn; 2 Key Laboratory of Solid Waste Treatment and Resource Recycle, Ministry of Education, Southwest University of Science and Technology, Mianyang 621010, China; qinshuai_t@163.com (Q.S.); lihan1099599@163.com (H.L.); ijiongyxu4159@163.com (Q.D.); GyanXXG123@163.com (Y.G.); chengchunjie1207@163.com (C.C.); 3 School of Resources and Safety Engineering, Central South University, Changsha 410000, China

**Keywords:** geopolymer foams, metakaolin, coal fly ash, physical properties, thermal conductivity, fire resistance

## Abstract

This paper aims to investigate the influence of coal fly ash (CFA) addition on the fireproof properties of the metakaolin-based geopolymer foams. The physical properties, thermal conductivity and fire resistance of the CFA-metakaolin-based geopolymer foams are discussed. The CFA-metakaolin-based geopolymer foams achieve a dry density between 259.43 kg/m^3^ and 349.73 kg/m^3^, a porosity between 71.78% and 72.98%, a thermal conductivity between 0.0871 W/(m·K) and 0.0944 W/(m·K) and a compressive strength between 0.38 MPa and 0.56 MPa, exhibiting better physical properties than that of the porous blocks without CFA addition. It is also found that the CFA addition could decrease the viscous sintering temperature and change the phase compositions of sintering products, resulting in the porous structure deterioration in a certain extent and obvious rise of the final reverse-side temperature during the fire-resistance tests. Fortunately, the conversion of the amorphous geopolymer gel to ceramics has helped to maintain the main skeleton structure stability. The CFA-metakaolin-based geopolymer foams still exhibit excellent fire resistance, and the reverse-side temperatures are always within 250 °C after 3 h fire-resistance tests.

## 1. Introduction

Porous materials have been widely considered in the field of fire protection due to their low density, low thermal conductivity, and rapid installation [1,2]. However, organic porous materials usually exhibit poor fireproof performance, and even produce toxic gases in the process of combustion, which has caused a number of fire accidents in China [3,4]. The application of traditional inorganic fire insulation materials, such as asbestos, mineral cotton, aerated concrete, may confront the problems of significant equipment investment, high energy consumption, and environmental pollution [4,5]. Inorganic lightweight porous materials, especially geopolymer foams (GFs), have attracted more and more attention owing to their light weight, non-toxic, and excellent high temperature resistance [6,7,8].

Geopolymer, firstly proposed by Davidovits in the 1970s, is an amorphous three-dimensional network structure gel [9], which is produced by synthesizing pozzolanic compounds or aluminosilicate source materials with highly alkaline solutions [10]. With the advantages of high thermal stability, excellent freeze-thaw resistance, low production cost and CO_2_ emissions, geopolymers seem very suitable to prepare inorganic foam materials and show a promising future in the field of fire protection [11]. Factually, metakaolin (MK)-based GFs exhibit remarkable fire-resistance, owing to the conversion of the amorphous MK-based geopolymer gel to ceramics during the fire-resistance tests [12,13]. However, the mechanical strength of MK-based geopolymer foams is lower than that of aerated concrete or foam ceramics with the same bulk density. The MK-based GFs with low bulk density usually signify low mechanical strength, which could possibly restrain their wide application. Many studies have been conducted to improve their mechanical strength. Some studies focus on the relationship between the pore structure and the mechanical properties of foam materials, showing that the mechanical behavior of the material is mainly influenced by the air-voids [14,15,16]. Meantime, researchers found that the dry density, water content, curing conditions and foaming agents can also affect the compressive strength by discussing various geopolymer formulations [17,18]. In fact, investigations on improving the strength of geopolymers have never stopped.

Coal fly ash (CFA) is a by-product from coal-fired power plants with excellent pozzolanic activity [19,20], which is widely proposed as a geopolymer precursor and has great potential to modify the mechanical strength of MK-based geopolymer foams. Meanwhile, characteristics of the CFA with spherical and fine particles, and lower requirement during the hydration reaction would increase its dispersibility and workability in MK-based geopolymer gel [21,22], leading to the obvious improvement in the rheological property of geopolymer slurry. The improvement in the rheological property of geopolymer slurry could effectively promote the hydration reaction extent, and help to achieve more a homogeneous pore structure, which is beneficial to the mechanical strength development of MK-based geopolymer foams. In addition, the abundant hollow microsphere in CFA may be conducive to increase the thermal insulation performance of GFs [23]. All the above features make the CFA a strong possibility to enhance the mechanical strength of MK-based GFs.

In this work, CFA addition is selected to modify the properties of the MK-based GFs. Influence of CFA addition on physical properties and fire resistance of the CFA-MK-based GFs has been deeply discussed.

## 2. Experimental

### 2.1. Materials

The MK was prepared by Kaolinite (Shanghai, China) and the CFA was collected from the dedusting system of Jiangyou Power Station (Sichuan, China). The particle size distributions of CFA were determined by Beckman Coulter LS13320 laser diffraction analyzer (D_50_ = 17.69 μm, D_90_ = 68.66 μm). Na_2_O_2_ (analytical reagents, Tianjing, China) was used as a chemical foaming agent, while calcium stearate (CaSt, analytical reagents, Tianjing, China) was used as a foaming stabilizer. The alkali activator was obtained by mixing sodium silicate solution (analytical reagents, Guangzhou, China) and sodium hydroxide (analytical reagents, Guangzhou, China). To study the influence of CFA addition on GFs, CFA was added at a mass fraction of 0%, 3%, 6%, 9% and 12%, respectively. The chemical compositions of MK and CFA are analyzed by X-ray fluorescence (Panalytical Axios, Almero, The Netherlands) and given in Table 1.

### 2.2. Sample Preparation

**Figure 1:**
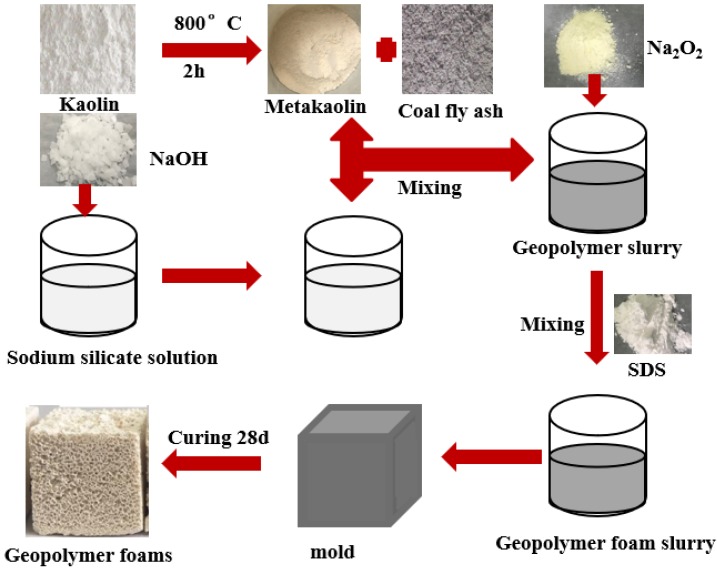
Preparation process of the CFA-MK-based GFs.

The preparation process of the CFA-MK-based GFs is shown in Figure 1. The weighed metakaolin, CFA, alkali activator and water was premixed for 3 min to give complete homogenization. Afterwards, Na_2_O_2_ was added to the mixture and mixed for another 10 s. Finally, the mixture was poured into molds having dimensions of 40 mm × 40 mm × 40 mm or 40 mm × 40 mm × 160 mm. The samples were then cured for 24 h at room temperature. After being removed from the molds, the samples were subjected to curing at room temperature for an additional 27 days. The CFA was added at a mass fraction of 0%, 3%, 6%, 9% and 12%, respectively. Mix designs of the CFA-MK-based GFs are in Table 2.

### 2.3. Physical Properties

The dry bulk density was measured by the ratio of the geometrical volume and weight. The mechanical strengths (compressive strengths and flexural strengths) were measured using a mechanical strengths testing machine (CMT5504, Shanghai, China). Porosity was determined based on ASTM C642-13. A 40 mm × 40 mm × 15 mm sample was selected to determine the thermal conductivity of GFs (TC 3000E, Xia Xi technology, Xi’an, China). The final results were obtained by recording an average of six specimens.

### 2.4. Fire-Resistance

**Figure 2:**
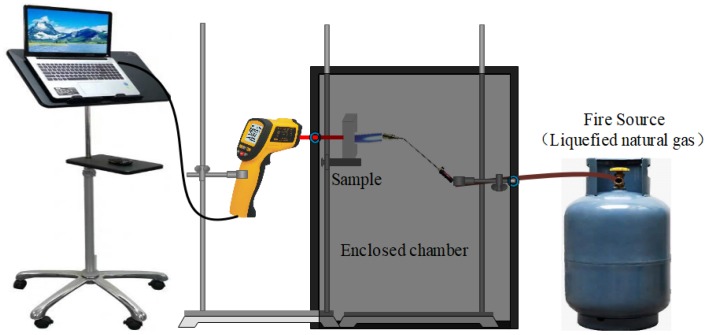
The setup for fire-resistance test.

Figure 2 shows the setup for the fire-resistance test. During the test, a selection of 20 mm-thick GFs were exposed to a 1100 °C flame, and the back-side temperatures of the specimens were determined by an infrared thermometer (Xima AR882+, Dongguan Wanchuang electronic manufacturing co. Ltd., Dongguan, China).

### 2.5. Microstructural and Mineralogical Characterization

The CFA-MK-based GFs were examined by X-ray powder diffraction (XRD) analysis (PANalytical B.V., Almelo, The Netherlands). The microscopic structure of the specimens was obtained by scanning electron microscope (SEM, Carl Zeiss AG, Jena, Germany).

## 3. Results and Discussion

### 3.1. Physical Properties

**Figure 3:**
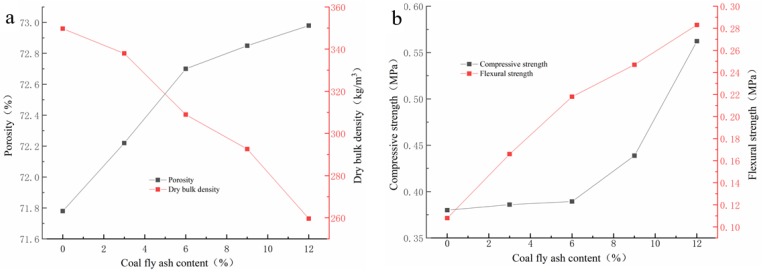
The trend of physical properties versus the addition of CFA: (**a**) porosity; (**b**) compressive strength.

As Figure 3a depicts, the porosity of GFS increases along with an opposite trend in dry density via the addition amount of CFA increases. With the increase of the CFA addition amount, the dry bulk density of GFs declines from 349 g/cm^3^ and 259 g/cm^3^, while the porosity increases from 71.78% to 72.98%. Due to the low water requirement of CFA for geopolymerization and the excellent dispersion of CFA particles in the geopolymer slurry, the CFA addition improves the fluidity and reduces the viscosity of geopolymer slurry. The change in the characteristics of the geopolymer slurry could help the gas generated by the foaming reaction to more easily diffuse into the whole slurry and promote the porosity of the hardened blocks [24]. The increase in porosity of geopolymer foams is usually accompanied by a decrease of the dry bulk density. When the CFA addition amount reaches to 12%, the dry bulk density is the lowest, which is lower than that of other reported studies [25]. However, once the addition percentage of CFA is more than 12%, the final setting time of geopolymer slurry is significantly extended, and it is difficult for geopolymer slurry to harden in this investigation.

It can be also seen from Figure 3b that mechanical strengths rise gently with the addition of CFA. With the increase of the CFA addition amount, the compressive strength and flexural strength increase from 0.38 Mpa to 0.5623 Mpa and 0.108 Mpa to 0.283 Mpa, respectively. Unlike other research, which suggested that the mechanical strength usually decreases with the increase of porosity [26,27,28], the porosity increase of the GFs has not had a negative impact on the strength development in this investigation. This is mainly owing to the following two reasons: (1) the high pozzolanic activity of CFA would be helpful to improve the mechanical strengths of MK GFs [29,30,31,32]; (2) the CFA addition could improve the rheological property of geopolymer slurry and help to form the porous geopolymer blocks with more uniform porous structure [24], which may be beneficial to the mechanical strengths development [33,34].

### 3.2. Thermal Conductivity

**Figure 4:**
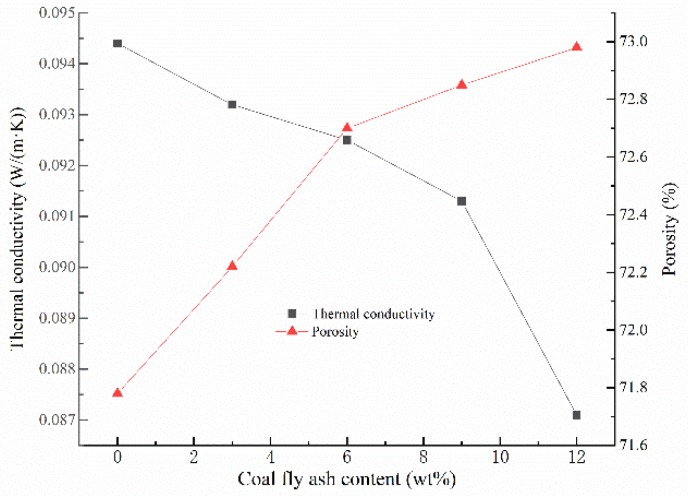
Thermal conductivity and porosity versus the addition of CFA.

Generally speaking, the thermal conductivity of GFs is mainly related to the materials porosity and pore distribution [28,35]. As can be seen from Figure 4, when the addition amount of CFA increases from 0% to 12%, the thermal conductivity of GFs declines from 0.0944 W/(m·K) and 0.0871 W/(m·K), which is close to the results in the present study [36,37]. In fact, the increase of porosity reduces the volume of the skeleton structure, and the thermal conductivity of it is much higher than that of the air within the voids, resulting in a higher thermal insulation effect of the whole GFs [38,39]. Meantime, the addition of CFA improves the rheological property of the geopolymer slurry and makes the pore distribution in the hardened blocks more uniform, which leads to a reduction in the thermal conductivity of GFs. In addition, the hollow microsphere in CFA can also promote the thermal insulation performance of GFs [23]. Considering the non-flammable character and contribution to environment, GFs could be strong candidates for non-load-bearing insulation structures.

### 3.3. Fire Resistance

**Figure 5:**
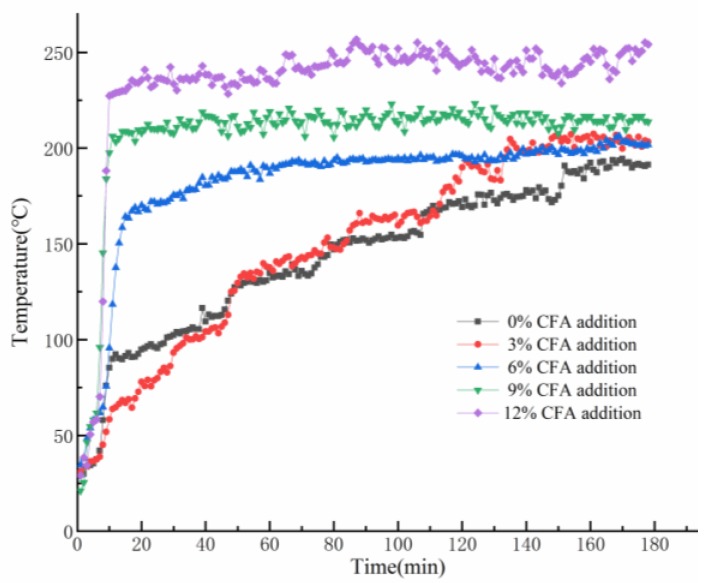
Variation trends of the back-side temperatures of the prepared GFs during the fire-resistance tests.

As it is shown in Figure 5, the variation of the back-side temperatures of GFs can be divided into two stages. At the beginning, dehydration of the prepared samples appears and make some irreversible microcracks in inner structure, which would cause the deterioration of thermal insulation performance and a dramatic rise of the back-side temperatures. Unexpectedly, the skeleton structures of the GFs have not collapsed, but still maintain stability at posterior high-temperature exposure. Meanwhile, the back-side temperatures of the GFs stop dramatically, increasing and constantly fluctuating in a very narrow range during the later period. As MK-based geopolymer gel would be easier to fuse and difficult to avoid structure deterioration when it is exposed to a 1100 °C flame [40], CFA partially substitutes MK in GFs synthesis and would bring an apparently negative impact on the thermal insulation performance. In fact, it can be found that the higher the CFA content is, the higher the final reverse-side temperature will be. Eventually, owing to the stable skeleton structure under the high temperature exposure, the geopolymer foams exhibit excellent fire resistance, and the back-side temperature of all the specimens are always within 250 °C.

### 3.4. XRD and XEM Analysis

**Figure 6:**
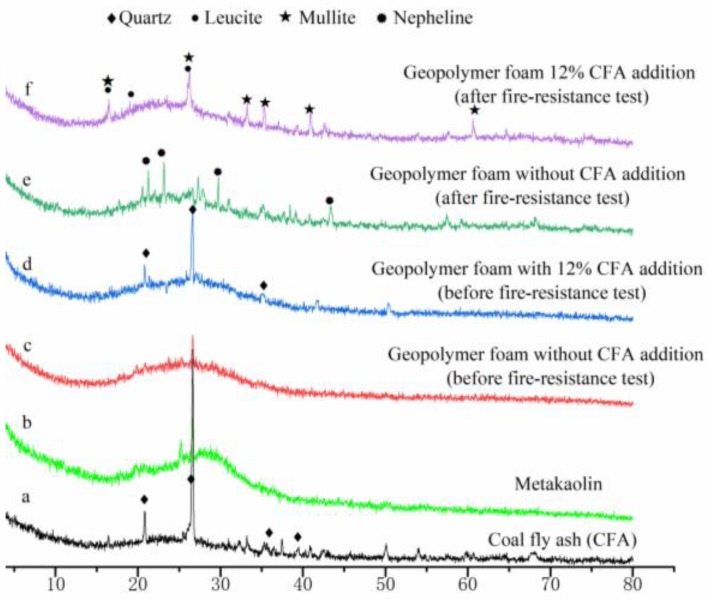
XRD patterns of the raw material and selected geopolymer foam specimens.

XRD patterns of the raw materials, selected geopolymer foam specimens before and after fire-resistance test are presented in Figure 6. Before the fire-resistance test, the main phase in GFs is amorphous geopolymer gel (Figure 6c,d). Meanwhile, quartz is detected in both CFA and geopolymer foam specimens with 12% CFA addition (Figure 6a,d). After the fire-resistance tests, nepheline has been found in the XRD pattern of the geopolymer foam specimen without CFA addition, while mullite and leucite appear in the geopolymer foam specimen with 12% CFA addition. The transformation of the amorphous skeleton materials to ceramic phases is beneficial to maintain porous-structure stability and excellent thermal insulation performance of GFs under a high temperature. The difference in ceramic phases between Figure 6e,f results from the addition of CFA: (1) the composition of GFs has been changes by CFA addition; (2) the viscous sintering temperature of the geopolymer matrix could be reduced, owing to the impure chemical components and lower melting point of the CFA.

**Figure 7:**
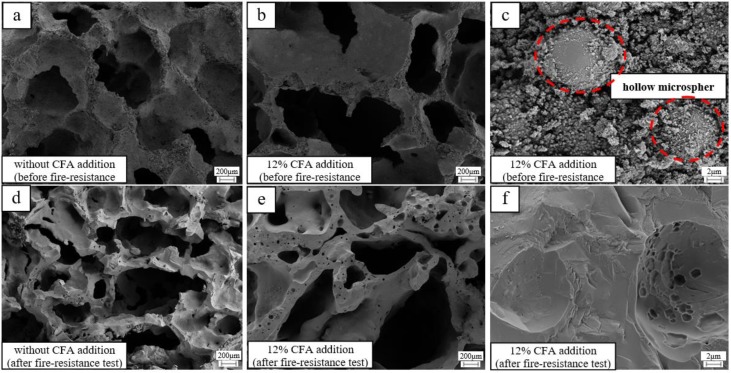
The micromorphology of selected geopolymer foam specimens: (**a**) without CFA addition, before the fire-resistance test; (**b**) 12% CFA addition, before the fire-resistance test; (**c**) 12% CFA addition, before the fire-resistance test; (**d**) without CFA addition, after the fire-resistance test; (**e**) 12% CFA addition, after the fire-resistance test; (**f**) 12% CFA addition, after the fire-resistance test.

The micromorphology of the GFs (samples with 12% and without CFA addition) before and after the fire-resistance test is given in Figure 7. Before the tests (Figure 7c), the CFA hollow microspheres are closely combined with the amorphous geopolymer gel, which is beneficial to reduce the dry density and thermal properties of the GFs. After the fire resistance tests, the surface of the prepared specimens transfers distinctly from rough and amorphous gel to smooth and crystalline structure. Moreover, CFA hollow microspheres disappear completely (Figure 7c,f), and plenty of smaller pores come out in the skeleton material (Figure 7d,e), indicating that the CFA or CFA-based geopolymer gel in the prepared geopolymer foam specimen have been melted when exposed to a 1100 °C flame. The CFA addition or CFA-based geopolymer gel with lower melting point may decrease the viscous sintering temperature and bring a negative impact on the porous structure of the hardened blocks.

In fact, the pore structure of the geopolymer foam specimen with CFA addition has been more serious shrinking and appears more inhomogeneous after the fire-resistance test, leading to the obvious rises of the final reverse-side temperature. Fortunately, the conversion of MK-based geopolymer gel to ceramics has helped to maintain the main skeleton structure stability of the GFs with the CFA addition. GFs with the CFA addition still exhibit excellent fire-resistance, and the back-side temperatures are always within 250 °C after the fire-resistance test.

## 4. Conclusions

In this work, the CFA-MK-based GFs were successfully fabricated and the influence of CFA addition on the dry bulk density, porosity, mechanical strengths, thermal conductivity and fire resistance of the CFA-MK-based GFs has been investigated. The improvement for the physical properties of the GFs is mainly due to the physical and chemical properties of CFA. As the addition amount of CFA varies from 0% to 12%, the porosity, mechanical strengths of the MK-based geopolymer foams have been promoted in a certain extent, while the dry density and thermal conductivity decrease simultaneously.

Although CFA is conductive to decreasing the thermal conductivity under ambient conditions, CFA addition would also decrease the viscous sintering temperature and change the phase composition of sintering products, leading to the porous structure deterioration of the GFs during the fire-resistance tests. Moreover, the pore structure deterioration has brought negative impact on the thermal insulation performance of the GFs, resulting in the obvious rise of the last back-side temperature during the fire-resistance tests. Finally, the change of the amorphous geopolymer to ceramics has helped to maintain the main skeleton structure stability. The CFA-MK-based GFs still show excellent fire resistance, and the back-side temperature is always within 250 °C three hours after the fire-resistance test.

## Figures and Tables

**Table 1:** Chemical compositions of MK and CFA (mass%).

Materials	SiO_2_	Al_2_O_3_	Na_2_O	MgO	Fe_2_O_3_	CaO	K_2_O	TiO_2_	SO_3_	Others
MK	57.24	37.97	0.24	0.14	0.97	0.14	1.20	0.74	0.57	0.79
CFA	44.17	15.33	0.38	0.54	13.70	17.25	3.36	2.19	1.57	1.51

**Table 2:** Mix design of the CFA-MK-based GFs.

MK (g)	CFA (g)	Alkali Activator (g)	Na_2_O_2_ (g)	Calcium Stearate (g)	Distilled Water (mL)
84.00	0 (0%)	56	3.640	0.364	42.0
81.48	2.52 (3%)	56	3.640	0.364	39.6
78.96	5.04 (6%)	56	3.640	0.364	37.2
76.44	7.56 (9%)	56	3.640	0.364	34.8
73.92	10.08 (12%)	56	3.640	0.364	32.4
